# The Medical Calendar; or Student's Guide to the Medical Schools, &c.

**Published:** 1829-07-01

**Authors:** 


**THE MEDICAL CALENDAR; OR, STUDENT'S GUIDE TO THE MEDICAL SCHOOLS IN EDINBURGH, LONDON, DUBLIN, &c. TOGETHER WITH THE REGULATIONS OF THE PUBLIC BOARDS, CORPORATIONS, &c. IN GREAT BRITAIN AND IRELAND. Small Octavo, pp. 220. Price Four Shillings. 1829.**

MEDICAL SCIENCE is now become an article of such extensive commerce, and the manufacture of it a trade so very profitable, that manufactories and depôts are daily multiplying throughout these islands. It was therefore high time that a calendar should be annually constructed to direct the public to the best as well as the cheapest shops where this valuable commodity may be obtained in its greatest purity.

Who can look back through memory's telescope for 30 or 40 years, and not be convinced that modern REFORMERS, the Peels and Warburtons of the day, have "broken in upon the constitution of 1798," and threaten us with ruin! There was a time, and many of our readers must remember it with a sigh, when the anatomico-chirurgical manufactory in Blenheim Street alone could furnish the Army, Navy, and East India Company—that is to say, the whole of the exportation trade (besides a considerable proportion of home consumption) with DOCTORS—and that, too, with as much celerity and cheapness, as *Brummagem* furnished muskets for the same services! Who can pass up that renowned street, without missing those balmy odours on which his olfactories were wont to regale, while he lingered on the steps that led him into the "tide of human existence," for ever rushing along Oxford "Road?"

"These were thy charms—but all these charms are fled."

That great emporium of anatomico-chirurgery is now shattered into atoms, and a host of petty markets, or rather stalls are set up, in every direction, for the retail of little "odds and ends" of anatomy, garnished with much preceptive surgery that passes through many *heads* before it reaches any *hands!* This is the "march of intellect." Be this march slow or quick, we suspect that better anatomists were turned out of Windmill Street and Blenheim Street 30 years ago, than at the present period.* We are willing to hope that the difficulty of procuring subjects, and the expense of purchasing them, have been the main causes of this falling off in anatomical knowledge—but we cannot help fearing that there is less of professional enthusiasm, and more of pecuniary calculation, now than then.

* Mr. Thomas, of Leicester Place, who was then demonstrator of anatomy in Windmill Street, can vouch for the following fact. Some 28 or 30 years ago, six individuals, of whom the Editor of this Journal was one, formed themselves into a society, and each, in his turn, gave a public demonstration in anatomy daily at his own expense, in the theatre or dissecting-room. They not only did so, but invited the audience, who were very numerous, to discuss the merits of their demonstration, when it was over. Is there such zeal exhibited at the present day?

Considering, however, the increased influx of young men into the profession, in modern times, and the obstructed outlets for them, in these piping times of peace—taking into consideration the "res angusta domi," under which they embark, and the heavy expenditure which they must *unavoidably* encounter, we do not much wonder at the economy which they evince in all matters where they have a choice.

We are credibly informed by the principal medical booksellers who come most in contact with students, that compendiums and short cuts to knowledge are now almost the only works which they purchase, while the standard productions of medical and surgical science are left to moulder on the shelves! These are bad "signs of the times." The minds of medical students have been poisoned for some years past. They have been prompted to hate and despise their masters—to contemn the institutions before which they are to appear for examination—and they are taught to believe that it is a great merit to elude the laws by every species of artifice! But they have at length become acquainted with the nature of the poison, and they have dashed it from their lips. A better æra is approaching—and, when the facilities for prosecuting anatomy shall have been effected—a more uniform system of elementary education established—more rigid and practical examinations enforced—and clinical lectures delivered in all our hospitals, we have no doubt that the medical youth of this country will go forth to the practice of their profession better qualified than their forefathers.

But to return to the Calendar. It is mortifying to see in these limited isles so many places for granting degrees, diplomas, and licences in medicine and surgery, no two of them enjoining a similar code of elementary education! This is a sad state of things—but while it lasts, a calendar similar to that before us is indispensable. To give any analytical review of such a work would be impossible. We have looked over it, and we have reason to think that great pains have been taken to render it correct in all essential points of information. We shall present two or three articles which will, at the same time, afford specimens of the work, and useful intelligence to many who are educating their sons for the profession.

### I. Dublin.

*Regulations of the University of Dublin, relating to the Degrees of Bachelor and Doctor of Medicine.*

"For the Bachelor's Degree.—The Candidate must present to the Register of Trinity College Certificates of having attended Lectures on the following branches, viz. Anatomy, Surgery, Chemistry, Botany, Institutes of Medicine, Practice of Medicine, Materia Medica, and two courses of Clinical Lectures given in immediate succession. These Certificates must all be taken from the Professors in Trinity College, and the King's Professors, appointed by the Act for establishing a complete School of Physic in Ireland, unless some three Certificates for the same Courses in the University of Edinburgh be produced, which are admitted by the Board: But the Certificates from any other University or College, or from any private School, are not received as qualifications.

"On the production of the above Certificates by the Register, the Board grant commission to the Candidate to be examined by the four Medical Professors of the University, viz. the Regius Professor of Physic, the Professor of Anatomy, the Professor of Chemistry, and the Professor of Botany, provided the proper time, according to the statutes, has elapsed from the period of his graduating as a Bachelor of Arts, which examination is conducted in the Latin language. If the Candidate be found qualified, the Professors individually certify the same, on which he is permitted to perform the exercises mentioned in the statutes, and at the next half yearly commencement, the degree of Bachelor of Medicine is conferred on him by the Vice-Chancellor of the University.

"If the degree of Master of Arts be taken previously to that of Bachelor of Medicine, the degree of M. B. can be obtained in either the University of Oxford or Cambridge, which qualifies the person for being elected a Fellow of the College of Physicians of London, to whom several privileges and advantages belong;

"FOR THE DOCTOR'S DEGREE.—After the proper time, as specified in the statutes of Trinity College, the degree of Doctor of Medicine is obtained on submitting to a second examination by the Medical Professors of the University, writing and publishing a Latin thesis on some medical subject, and performing the exercises directed in the statutes for the degree of M. D.

"According to the Rules of the English and Dublin Universities, a portion of the study in Arts, as in Medicine, may be taken in one and finished in either of the other two Universities.

"Deposit for M. B. L.11, 15s—for M. D. L.22.

"The degrees of Bachelor and Doctor of Medicine, from Trinity College, rank with the degrees of M. B. and M. D. of Oxford and Cambridge." 143.

II. PARIS.

"At Paris the Medical Lectures are given, 1. By the Professors of the Faculty of Medicine at the School of Medicine ; 2. By Private Teachers, who have established Theatres around the School of Medicine. The Winter Courses continue from the beginning of November to the end of March, the Summer Courses from the beginning of April to the end of August. In September and October there are no Lectures."

"To the Parisian Hospitals, with the exception of the Hôtel-Dieu, the Venereal Hospital, and the Military Hospitals, there is almost unrestricted admission. Students who have been two years engaged in the study of medicine, and those who have obtained a Medical Degree or Surgeon's Diplomas, obtain tickets of admission to the Hôtel-Dieu, on applying to M. Dupuytren, the Surgeon.

"The *carte* which is obtained on application to the *Bureau* of the School of Medicine, gives the right of admission to the other Hospitals, as well as to the Lectures of the Professors of the School. This *carte*, unless a certificate is desired, such as is required for a degree either at Paris or elsewhere, costs nothing. Those who wish to obtain such certificates, with a view to graduate out of France, pay about 25 francs at every inscription, that is, every three months. Students who design to graduate at Paris pay about 50 francs at each inscription ; and the sums so paid are reckoned as part of the 1000 francs, which is the total expense of a degree at Paris, and of the studies in Medicine required for it.

"Beyond this fee at the inscriptions, the Student is at no expense in the public courses of the School of Medicine, unless for Subjects in the Dissecting-rooms. In the Dissecting-rooms at the Hôtel-Dieu, which are free to all, subjects cost from 6 to 8 francs. On paying about 60 francs, the Student may become a pupil of the Assistants in the Practical School; for this sum, eight bodies are allowed to six pupils.

"The LIBRARY of the FACULTY of MEDICINE is open for the consulting of books on Mondays, Wednesdays, and Fridays, from 11 to 3 o'clock.

"There are many Private Reading-rooms in Paris, where Students, on paying a small sum, may consult Medical Books daily. The Books are not usually lent out.

"The MUSEUMS of the SCHOOL of MEDICINE contain a magnificent Collection of Anatomical Preparations and Casts, Models of Surgical Instruments, and Specimens of the Articles of the Materia Medica. They are open on the same days, and at the same hours, as the Library.

"The BOTANIC GARDEN, or *Jardin des Plantes*, is one of the richest in the world. There are attached to it Collections of Animals, and other objects of Natural History. Lectures are given here in various departments of Natural Science.

"The FACULTY of MEDICINE at Paris confer two Degrees—the Degree of Doctor in Medicine, and that of Doctor in Surgery. Sixteen inscriptions, or four years' study, are required for either Degree. The Candidates must have obtained previously the Degrees of Bachelor of Letters and of Bachelor of Sciences. There are five examinations for the Degree of Doctor, which take place, by the late regulations, at stated periods throughout the four years' course of study. A Thesis also is defended, which is afterwards printed." 161.

### III. GLASGOW.

"MEDICINE.—I. Every Candidate for a Medical Degree must bring evidence that he has reached the age of Twenty-one.

"II. He must bring evidence of having attended for four years some University in which Medicine is regularly taught, or the Lectures delivered in the Theatre of the College of Surgeons, Dublin; or those delivered in London: one of these years, at least, he must have attended the University of Glasgow.

"III. He must produce Certificates of having attended the following Medical Classes, in the above mentioned Schools—each Course being reckoned to last during six calendar months, viz.

"Anatomy, during Two such Courses.
"Chemistry, during Two such Courses.
"Institutions of Medicine, One such Course.
"Practice of Medicine, One such Course.
"Materia Medica, One such Course.
"Midwifery, One such Course.
"Surgery, One such Course.
"Botany, One Course in a University.
"Infirmary, during Twelve Months.

"N. B.—Two London Courses, of between three and four months each, to be reckoned equivalent to one six months' Course.

"IV. Each Candidate for a Medical Degree must announce his intention, and lodge the requisite Testimonials with the youngest Member of the Medical Faculty in the University, two months before the time of Graduation; that is to say, by the 1st of March and the 1st of June, otherwise he cannot be taken on Trials till the following year.

"V. Every Candidate for a Medical Degree shall undergo three Examinations. The first on Anatomy and Physiology; the second on Chemistry and Pharmacy; and the third on the Practice of Medicine. He shall likewise write a Latin Commentary on an Aphorism of Hippocrates, and a Medical Case.

"VI. The Degrees shall be conferred on those Candidates who have acquitted themselves to the satisfaction of the Examinators, on the last Wednesday of April, and the first Wednesday of August, in each year, and at no other time." 174.

### IV. ABERDEEN.

MEDICAL DEGREES.—"I. Every person offering himself as a Candidate for the Degree of M. D. shall first produce satisfactory evidence of his possessing a good character, and of his having attained the age of twenty-five years; and secondly shall lay before the Senatus Academicus, Certificates of his having obtained the

Degree of A.M. in this, or some other University, after the usual Examinations;* of his having attended Courses of Lectures on Anatomy, Surgery, Chemistry, Materia Medica, Theory and Practice of Physic, and Botany, in this or some other University or celebrated School, under Professors or Teachers of reputation; and of his having attended, for three or more years, a Medical Hospital containing the average number of at least eighty patients.

"II. After the Senatus Academicus shall have been fully satisfied on the above preliminary points, the applicant shall be received as a Candidate for the Degree of M. D. and shall be required to appear before the University at one of their stated terms for granting such Degrees, and in their presence be examined by the Medical and other Professors on the different branches of Medical Science—on his knowledge of the Greek and Latin languages—and on such other branches of Literature as they shall see proper. If fully satisfied with the qualifications of the Candidate, the University shall confer on him the Degree which he solicits." 180.

### V. ST. ANDREW'S.

MEDICAL DEGREES.—"*1st*, No Degree shall be conferred on an absent Candidate.

"*2d*, The Candidate must produce a satisfactory Certificate that he is upwards of 21 years of age, and that he is of unexceptionable moral character.

"*3d*, The Candidate, if he be not in possession of a Diploma as A. M., must produce Certificates of his having had a Liberal and Classical Education, and be ready to undergo an examination as to his proficiency in Classical Literature by a Committee of the University, or by the Senatus Academicus.

"*4th*, The Candidate must produce Certificates that he has attended in some University, or celebrated School of Medicine, for at least four complete Sessions, during four years, the following branches of Medical Education, viz. Anatomy and Surgery—Practical Anatomy, or Private Dissections—Materia Medica and Pharmacy—Chemistry and Chemical Pharmacy—the Theory of Physic—the Practice of Physic—Midwifery and the Diseases of Women and Children—Botany and Clinical Lectures delivered by Professors, or accredited Medical Officers, on the cases of the sick in a large public Hospital. (It is not meant that the Candidate shall have attended more than two or three of the above-mentioned Medical Classes during one Session.)

"*5th*, The Candidate must bring Certificates of his having attended the daily visitations of the Physicians and Surgeons of such Hospital for at least six months a-year during two different years.

"*6th*, If the Candidate shall have so far satisfied the University, the Rector shall be requested to fix a day for his examination by the Medical and other Professors on all the branches of Medical Science; and provided this examination shall satisfy the Senatus Academicus, the Candidate shall have delivered to him a Medical case or cases with questions subjoined, which cases he must answer in writing, and defend his answers before the Members of the University.

"*7th*, The whole proceedings shall then be submitted to the consideration of the Senatus Academicus; and if they are satisfied, the degree of M. D. shall be conferred on the Candidate by the Rector, in the Hall of the public Library.

"Such are the Regulations appointed by the University of St Andrew's to be observed in all ordinary cases. When, however, the Candidate, possessed of a Surgical Diploma from Edinburgh, London, Dublin, or Glasgow, shall have

"* As the requisition respecting the Degree of A. M. might prove injurious to some Medical Students whose education is now in progress, were it to take effect immediately, it is resolved that it shall not come into full force till the year 1830."

been in regular practice for some years, or when he shall have been appointed and have acted as Surgeon in his Majesty's Navy or Army, or to the Forces or Ships of the East India Company—a three years' attendance on the above-mentioned courses of Medicine, and one year's attendance on a public Hospital, shall be deemed sufficient to rank him as entitled to be examined.

"Farther, it is desirable that the Candidate communicate by letter with the Medical Professor, and send him a list of the Certificates he means to produce, before he repairs to St. Andrew's for examination. This may prevent disappointment, delay, and expence to the Candidate.

"*8th*, The University reserves to itself the power to confer Honorary Degrees in Medicine on highly distinguished individuals, who cannot be expected to comply with the above Regulations; but every such Honorary Degree shall be conferred unsolicited and free of expence.—*St. Andrew's, 25th Feb.* 1826." 186.

The foregoing specimens are sufficient. The work is extremely useful, and cannot fail to be encouraged.

